# The complexity of tackling multimorbidity in atrial fibrillation: how European projects are reshaping our approach to comorbidities

**DOI:** 10.1093/ehjopen/oeaf132

**Published:** 2025-10-27

**Authors:** Giulio Francesco Romiti, Tze-Fan Chao, Gregory Y H Lip

**Affiliations:** Department of Translational and Precision Medicine, Sapienza – University of Rome, Viale dell’Università 37, Rome 00185, Italy; Liverpool Centre for Cardiovascular Science at University of Liverpool, Liverpool John Moores University and Liverpool Heart & Chest Hospital, 6 West Derby Street, Liverpool L7 8TX, UK; Division of Cardiology, Department of Medicine, Taipei Veterans General Hospital, Taipei, Taiwan; Institute of Clinical Medicine, and Cardiovascular Research Center, National Yang Ming Chiao Tung University, Taipei, Taiwan; Liverpool Centre for Cardiovascular Science at University of Liverpool, Liverpool John Moores University and Liverpool Heart & Chest Hospital, 6 West Derby Street, Liverpool L7 8TX, UK; Department of Clinical Medicine, Aalborg University, Aalborg, Denmark; Department of Cardiology, Lipidology and Internal Medicine with Intensive Coronary Care Unit, Medical University of Bialystok, Bialystok, Poland

**This editorial refers to ‘Development of three-step holistic care pathways to detect and manage comorbidities in patients with atrial fibrillation: the Horizon 2020 EHRA-PATHS consortium’, by R. Önder *et al.*  https://doi.org/10.1093/ehjopen/oeaf120 and ‘Implementation of new concise care pathways into a software tool to detect and manage comorbidities in older patients with atrial fibrillation: the Horizon 2020 EHRA-PATHS project’, by R. Önder *et al.*  https://doi.org/10.1093/europace/euaf111.**

Multimorbidity is a growing issue in patients with atrial fibrillation (AF). Compared to two decades ago, epidemiological trends show that patients with AF present with an increasing number of comorbidities at diagnosis.^1^ In these patients, the burden of multimorbidity is not limited to cardiovascular conditions but extends to other non-cardiovascular diseases, which are more prevalent than in general, non-AF population.^2^

In recognition of the major contribution of multimorbidity on the trajectories of patients with AF,^3^ international guidelines now put significant emphasis on the need for holistic management, with specific efforts towards optimal treatment of comorbidities.^4,5^ Nonetheless, managing multimorbidity is challenging in clinical practice. Several factors hamper optimal management of comorbidities in patients with AF, including underdiagnosis (particularly of non-cardiovascular comorbidities^6^), limited time and resources to assess comorbidities, and lack of structured pathways to ensure timely and appropriate referral when indicated. The impact of these factors is bolstered for non-cardiovascular comorbidities, particularly when AF is managed outside multidisciplinary teams or specialized AF clinics.

Europe-wide, different projects aim to improve management and outcomes of multimorbidity in patients with AF.^7–9^ In two recently published article,^9,10^ Önder and colleagues present some of the central aspects of the EHRA-PAHTS project, that aims ‘to develop, implement and evaluate systematic care pathway to tackle multimorbidity in older patients with AF’. In the first paper,^9^ the authors describe the developmental process of 23 concise care pathways, each addressing a specific cardiovascular or non-cardiovascular comorbidity/condition; candidate comorbidities were selected according to their relevance in the context of AF. All 23 care pathways were based on a common template encompassing 3 steps (i.e. detection trigger, evaluation/confirmation, and start of management or referral, with key performance indicator), to be completed within six months from the first consultation. These care pathways were developed internally at the EHRA-PATHS consortium in a two-phase process; then, the care pathways underwent two rounds of a Delphi process, in which consensus on the proposed pathways was seek among consortium members and patient representatives. Pathways with <80% consensus at first round were adjusted and passed to a second round, after which the outstanding pathways with <80% consensus were further discussed with experts and finalized by the central coordinating centre. The approved pathways were subsequently implemented in a web-based software tool. The development of such tool is described in the second paper,^10^ along with the results of a survey on software evaluation and preliminary data on the time needed by AF nurses to complete the ‘detection trigger’ step of the care pathways (which was found lower than previously estimated by the survey’s respondents). The software will be central in the EHRA-PATHS randomized trial, which will involve 11 European countries, and will evaluate the care pathways.^9^

The developments of the EHRA-PATHS project described in the two articles by Önder *et al.* offer important lessons on the complexity of addressing multimorbidity in clinical practice, and on the challenges of developing a software to aid clinical management. Indeed, up to 23 ‘comorbidities’ were identified (some of which were conditions, such as low medication adherence and physical inactivity; and syndromes, such as frailty), being considered relevant in the trajectory of patients with AF, underlining how difficult is to assess and target multimorbidity in clinical practice. Moreover, the focus on ‘detection triggers’ as the first step of the care pathway appears rationale, considering that underdiagnosis of comorbidities is one of the actionable barrier to reduce the burden of multimorbidity and ensure holistic management of patients with AF. EHRA-PATHS also shows that development of softwares for clinical use poses unique challenges to clinical investigators, but is feasible if managed by an experienced team with clear goals and a structured approach. These lessons will be crucial for future researchers that will engage in the process of developing software for use in clinical practice.

On the other hand, it is important to note that comorbidities do not occur in isolation: they rather occur in combination and follow patterns and ‘phenotypes’,^11^ with heterogeneous association with prognosis.^12^ While the identification and optimal treatment of individual comorbidities is central in the management of AF, it is also important to acknowledge that ‘one size may not fit all’. Indeed, patients with multimorbidity have complex health needs, which are better served by a unitary and holistic approach that account for the complex and multi-directional interaction of comorbidities, the expected benefits and clinical priorities, and—most importantly—the need to tailoring approaches based on individual characteristics. This is particularly important in the context of ‘clinical complexity’—a common scenario in which multimorbidity occurs with ageing, frailty and polypharmacy, and that is associated with worse prognosis.^13^ For these patients, it is crucial to avoid care fragmentation, which increases with number of comorbidities and is associated with poor outcomes.^14^ Conversely, coordination of care should be pursued,^15^ in the context of a holistic and comprehensive management framework.

How to streamline such holistic approach to target multimorbidity in patients with AF? Clinicians should recognize that identification and treatment of comorbidities is one of the pillars constituting the fundamentals of AF management; this step should be part of a coordinated, unitary and integrated approach, encompassing thromboembolic risk prevention (recognizing that stroke and bleeding risks increases with multimorbidity^3^), and better control of AF-related symptoms. Over the years, international guidelines have proposed different patient care pathways to streamline such an integrated care approach to AF:^5^ these include the evidence-based ‘ABC’ pathway or other (untested) acronyms such as the ‘SOS’ and ‘CARE’ pathways, all built upon the three management pillars described above. Indeed, the efficacy of the ABC pathway in patients with AF has been validated in observational studies^16,17^ and in two cluster randomized trials^18,19^ and has been also shown in clinically complex patients.^13^ A third cluster randomized trial is currently ongoing in Europe, in the context of the AFFIRMO Horizon 2020 project,^7^ and is evaluating whether the integration of comprehensive geriatric assessment (CGA) along with the ABC pathway is associated with improved prognosis in patients with AF and multimorbidity.

In this scenario, the EHRA-PATHS and the AFFIRMO trials appear to target multimorbidity from two different, but complementary perspectives: EHRA-PATHS aims for structured comorbidity evaluation and management, through the implementation of systematic care pathways^9,10^ AFFIRMO, on the other side, will look at the efficacy of a comprehensive and integrated approach (the ABC pathway, in which comorbidity management is one important pillar, with integration of CGA) to manage the overall complexity of multimorbid patients with AF. Answers coming from the two trials have the potential to reshape the way we approach to multimorbidity in patients with AF. Future developments will also come from other ongoing projects which aim to integrate artificial intelligence approaches to personalize management of patients with AF.^8,20^ For the time being, multimorbid patients with AF should be managed according to an holistic approach, in which management of comorbidities is part of an integrated care pathway that starts with stroke risk prevention and better symptoms control, as recommended by the evidence-based ABC pathway, or other equivalent acronyms.
